# The Most Influential Medical Journals According to Wikipedia: Quantitative Analysis

**DOI:** 10.2196/11429

**Published:** 2019-01-18

**Authors:** Dariusz Jemielniak, Gwinyai Masukume, Maciej Wilamowski

**Affiliations:** 1 Department of Management in Networked and Digital Societies Kozminski University Warszawa Poland; 2 The Irish Centre for Fetal and Neonatal Translational Research Department of Obstetrics and Gynaecology University College Cork Cork Ireland; 3 Faculty of Economic Sciences University of Warsaw Warsaw Poland

**Keywords:** citizen science, medical journals, open knowledge, Wikipedia, knowledge translation, journalology, medical publishing, scholarly publishing

## Abstract

**Background:**

Wikipedia, the multilingual encyclopedia, was founded in 2001 and is the world’s largest and most visited online general reference website. It is widely used by health care professionals and students. The inclusion of journal articles in Wikipedia is of scholarly interest, but the time taken for a journal article to be included in Wikipedia, from the moment of its publication to its incorporation into Wikipedia, is unclear.

**Objective:**

We aimed to determine the ranking of the most cited journals by their representation in the English-language medical pages of Wikipedia. In addition, we evaluated the number of days between publication of journal articles and their citation in Wikipedia medical pages, treating this measure as a proxy for the information-diffusion rate.

**Methods:**

We retrieved the dates when articles were included in Wikipedia and the date of journal publication from Crossref by using an application programming interface.

**Results:**

From 11,325 Wikipedia medical articles, we identified citations to 137,889 journal articles from over 15,000 journals. There was a large spike in the number of journal articles published in or after 2002 that were cited by Wikipedia. The higher the importance of a Wikipedia article, the higher was the mean number of journal citations it contained (top article, 48.13 [SD 33.67]; lowest article, 6.44 [SD 9.33]). However, the importance of the Wikipedia article did not affect the speed of reference addition. The *Cochrane Database of Systematic Reviews* was the most cited journal by Wikipedia, followed by *The New England Journal of Medicine* and *The Lancet*. The multidisciplinary journals *Nature*, *Science*, and the *Proceedings of the National Academy of Sciences* were among the top 10 journals with the highest Wikipedia medical article citations. For the top biomedical journal papers cited in Wikipedia's medical pages in 2016-2017, it took about 90 days (3 months) for the citation to be used in Wikipedia.

**Conclusions:**

We found evidence of “recentism,” which refers to preferential citation of recently published journal articles in Wikipedia. Traditional high-impact medical and multidisciplinary journals were extensively cited by Wikipedia, suggesting that Wikipedia medical articles have robust underpinnings. In keeping with the Wikipedia policy of citing reviews/secondary sources in preference to primary sources, the *Cochrane Database of Systematic Reviews* was the most referenced journal.

## Introduction

Wikipedia, the multilingual encyclopedia, is the world’s largest and most visited online general reference website and, arguably, the largest collaborative project of humankind [1]. Wikipedia reflects the state of scientific knowledge but also shapes science; ideas that are integrated into the encyclopedia are used more in scientific journals [2]. There is evidently a feedback loop between Wikipedia and scholarly journals, which accelerates research. Indeed, traditional journals are increasingly citing Wikipedia formally [3], and the general distrust of Wikipedia in academic circles is decreasing [4].

Wikipedia is widely used by health care professionals and students as well as educators, journalists, and policy makers, among others [5,6]. In fact, medical students perform better on tests when they use Wikipedia as compared to standard medical digital textbooks (statistically significant difference) or a contemporary point-of-care medical website (statistically nonsignificant difference) [7]. The quality of information on Wikipedia on medical topics is generally high [8,9], although this quality is, admittedly, culturally influenced [10] and partly dependent on the editor’s experience [11], with varying article readability [12,13].

The inclusion of academic articles into Wikipedia remains a topic of interest for scholars from various fields [14]. However, it is still not entirely clear how often and quickly recent sources are used to support Wikipedia’s medical articles, from the point of publication to their incorporation into Wikipedia [15], and previous studies on the topic are limited in scope [16].

In this study, we analyzed 39,564 medical articles from the English-language Wikipedia to determine the time taken for journal publications to reach Wikipedia and to identify journal outlets that are most likely to be included in Wikipedia. We developed a ranking for medical journals based on their representation on Wikipedia. We hypothesized that the time taken for journal publications to reach Wikipedia was declining and that high-impact factor journals were represented more often.

## Methods

We analyzed the number of days between journal article publication and its citation in the English-language Wikipedia, treating this measure as a proxy for the information-diffusion rate. For our analysis, we selected 39,561 medical articles on Wikipedia that were marked as “medical” by the Wikipedia community [17] (as of October 10th, 2017).

It is worth mentioning that not all articles tagged as medical (~25%) were expected to cite the scholarly literature because of the topics they covered or the early stage of their development. Among other Wikipedia special pages, redirect (~9%), category (~9%), and template pages (2%) were not expected to have citations [17].

From 11,314 Wikipedia medical articles, we found citations to 137,889 articles from over 15,000 journals. We retrieved the dates on which the articles were added to Wikipedia and the date of publication from Crossref for 108,600 references using an application programming interface. In 8,384 of these references, the date of addition to Wikipedia preceded the official date of publication, which does not necessarily signify an error, but might refer to preprints (Multimedia Appendix 1).

## Results

**Figure 1:**
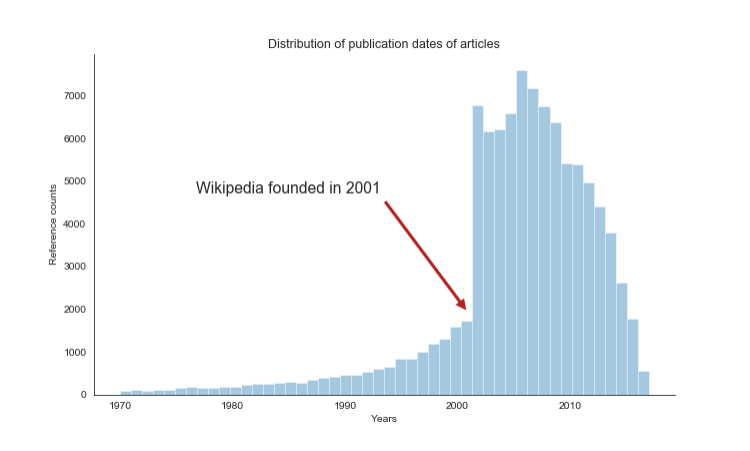
Journal articles cited in Wikipedia according to their year of publication.

When we analyzed the publication dates in the citations, we observed two important points. First, there was a clear increase in the number of articles published in or after 2002. Since Wikipedia was started in 2001, this finding is not surprising. Wikipedia editors have a clear preference for adding new sources (Figure 1), which is expected because “recentism” is an established phenomenon on Wikipedia, wherein coverage of recent events is disproportionately greater, and the Wikipedia community itself considers it a factor to be accounted for [18]. In the case of medical research, there is obvious value in focusing on more recent studies, as older studies may be obsolete. Nevertheless, the historical long view tends to be lost consequently. Second, we grouped the articles according to their importance decided by the Wikipedia community [17] and calculated the average number of citations per article (Table 1). An example of an article of highest (top) importance would be that on cancer (crucial to medicine). An abdominal pain article (directly affects many readers) would be of high importance; an abdominal mass article (interesting to many readers) would be of mid-importance, and an article on McBurney’s point, an anatomical point in the abdomen, would be of low importance (other articles of low importance include hospitals, very rare diseases, and individuals).

As expected, the higher the importance of an article, the more citations it contained on an average. For further analysis, we clustered articles of “top”, “high”, and “medium” importance together and considered them as “important” articles, because their total count was roughly similar to the number of articles of “low” importance. Only about half of the articles had citations to journal articles, which is consistent with previous research [5].

**Figure 2:**
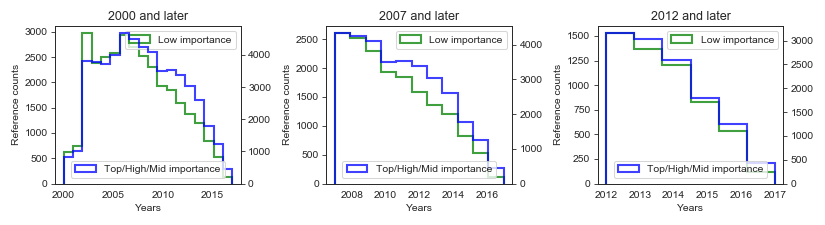
Histograms of the number of citations between the two quality groups.

We had two further working hypotheses. First, we assumed that highly important articles would be updated more often, and there would be a higher priority to update the references with the most recent research results. In the first two histograms in Figure 2, the blue line that represents the number of references for articles of higher importance is above the green line. However, in the third histogram both lines almost overlap, suggesting that before 2012, there was a higher priority for articles of higher importance. However, that was not the case after 2012, possibly due to equalization of maturity of articles in higher- and lower-importance groups or because this time period was not long enough for new research results that would require reference updating. Thus, after 2012, we did not observe a difference in the age distribution of the referenced articles. Second, we assumed that the more obscure articles are more likely to be developed by editors with a conflict of interest (COI), motivated to promote their own work on Wikipedia, who add references immediately upon their publication.

**Table 1:** The average number of references according to importance of the Wikipedia article.

Article importance	Articles, n (%)	References, mean (SD)	References, median (interquartile range)
Top	90 (0.8)	48.13 (33.67)	44.5 (17.25-67.5)
High	783 (6.9)	19.51 (22.21)	11 (5-26)
Medium	4532 (40.1)	9.39 (13.31)	5 (2-11)
Low	5905 (52.2)	6.44 (9.33)	3 (2-7)

If the articles of lower importance were prone to quick referencing by editors with COI, the green histograms in Figure 2 would be much flatter, especially in the new articles (after 2012). For the medical sciences, this was an important observation, because Wikipedia is not solely focused on developing new articles and does not discriminate among topics: References are added equally to the most popular and important articles and the most obscure articles.

**Figure 3:**
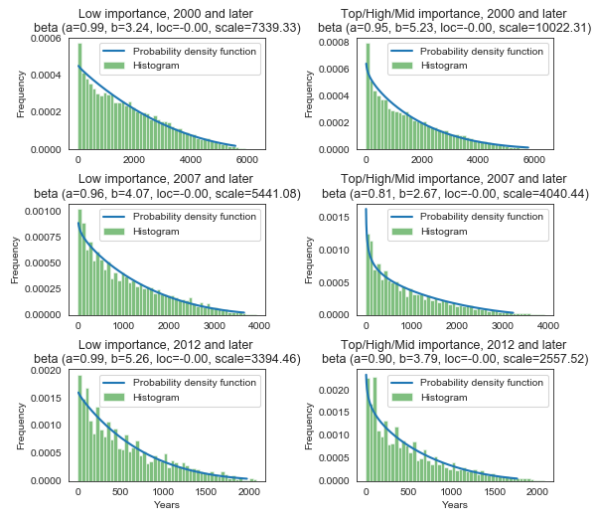
Estimated distributions of the number of days between publication and citation on Wikipedia.

**Figure 4:**
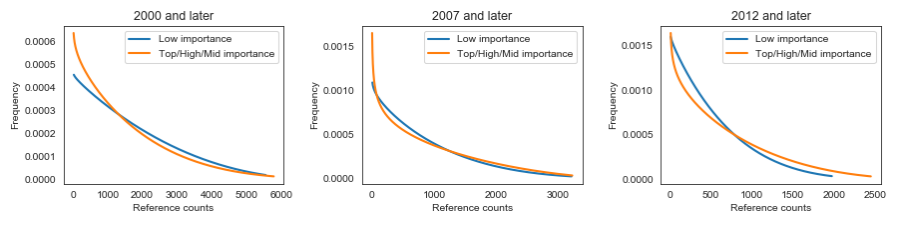
Comparison of the estimated distributions of the two quality groups.

As observed, the average age of a referenced article does not have to be correlated with the number of days between the journal article publication date and the date of its citation on Wikipedia. To verify our second hypothesis, we analyzed the data in detail. First, we plotted histograms for both importance groups in three time periods (2012 and later, 2007 and later, 2000 and later; Figure 3). The distribution was smooth and remained independent of the quality and importance of articles on Wikipedia. Independent of the time perspective and without focusing on just the important articles, the distributions were similar between both importance groups (Figure 4). For easier comparison, we overlaid the estimated distributions in Figure 4. In all cases, the empirical distribution was best described by a beta distribution. In every analyzed period, the estimated distribution had higher kurtosis for higher-importance articles. The differences in distributions may not be substantial, even if they are statistically significant. Thus, the time taken for breaking research to reflect on Wikipedia is a stable, reliable measure.

**Figure 5:**
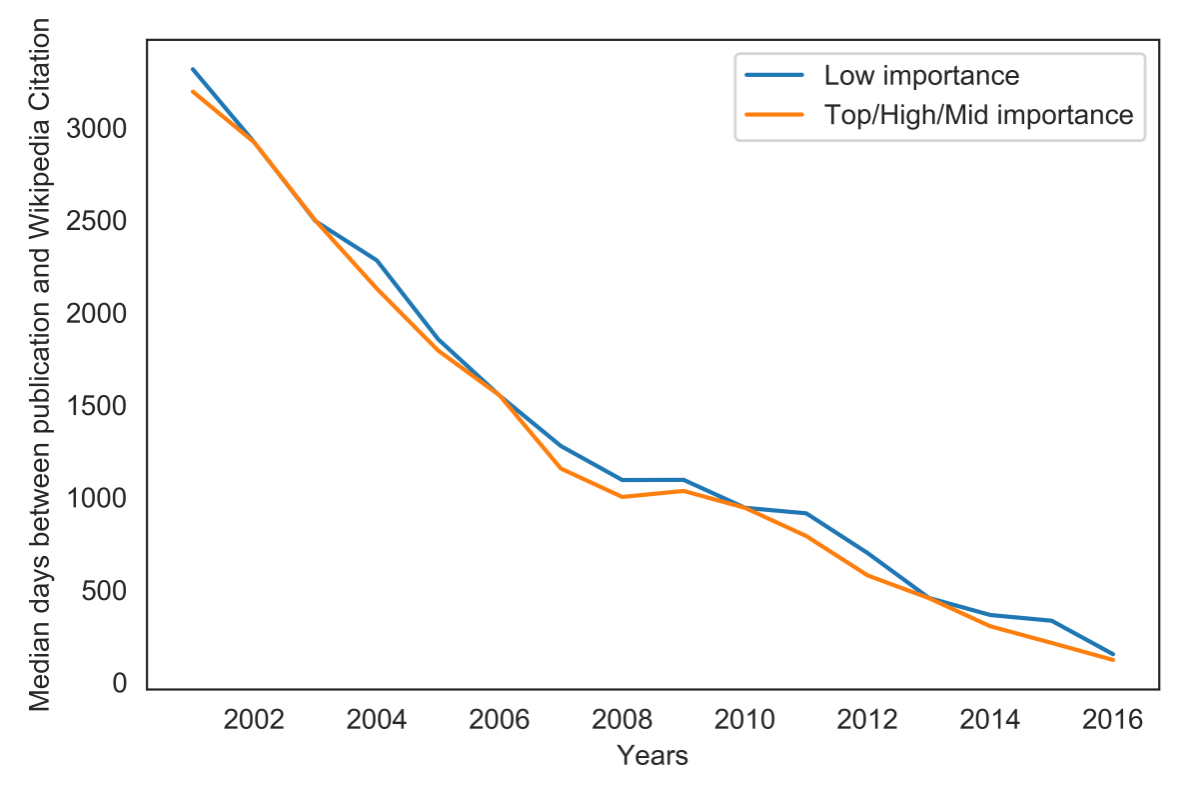
Change in average time between publication and Wikipedia citation over time.

We thereafter analyzed the change in time between publication and citation on Wikipedia over time and the difference between the two importance groups (Figure 5). We found that the time from journal article publication to incorporation into Wikipedia has been declining substantially from 2001, when Wikipedia was started. Addition of the historical canon explains the lengthy time to incorporation during the first few years of Wikipedia’s existence. In following years, as the canonical works were already covered, the time to incorporation in Wikipedia decreased. In 2016, the median time for articles of higher importance to be referenced in Wikipedia was 120 days; for articles of lower importance, 150 days; and for the 10 most highly cited journals, <90 days. As of mid-2018, it took about 3 months for articles published in high-impact journals to be incorporated into Wikipedia. Over time, with the median value calculated annually, it took up to 30% longer for articles of lower importance to be cited on Wikipedia as compared to articles of higher importance. Although in some years, there was little-to-no difference in the time to citation, the references of articles of higher importance in Wikipedia were updated faster than those of articles of lower importance.

**Figure 6:**
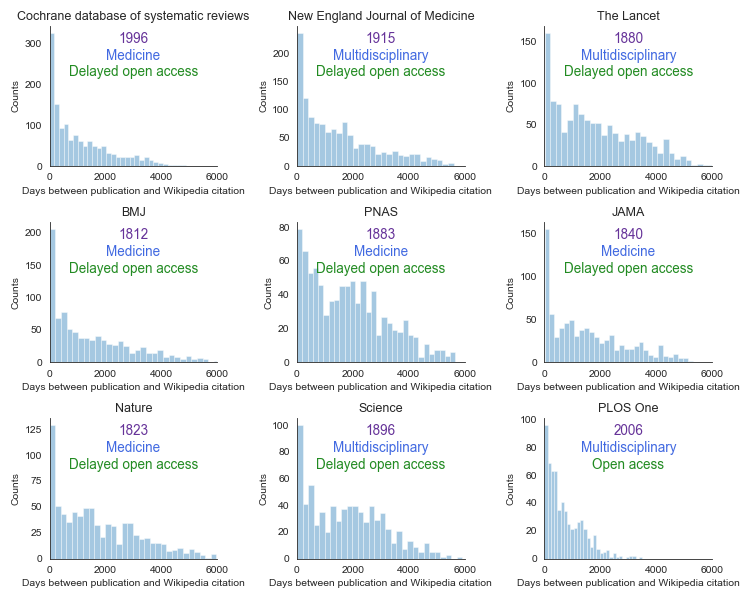
The number of days from journal article publication to Wikipedia citation for the top nine journals. Each graph includes the name of the journal, the year the journal was founded, and the type of journal access at the top.

Next, we analyzed the characteristics of the most cited journals of all analyzed articles. On comparing different periods, we observed relative stability in the rankings for most journals (Table 2), with two exceptions: *Proceedings of the National Academy of Sciences* (PNAS), whose position in the ranking dropped systematically over time, and *PLOS One*, which is characterized by a rising trend (Table 2). The change in the number of citations over time is clearly visible for PNAS (Figure 6). Furthermore, the number of citations for a given journal is characterized by spikes and sharp changes from year to year in some cases, which is only natural and may result from special issues on a topic, conferences coverage, or dedicated efforts of Wikipedia editors to cover specific topics. A list of the top 200 journal articles according to Wikipedia is presented in Multimedia Appendix 2.

To better understand the process of dissemination in Wikipedia, we calculated the time from publication to citation for individual journals (Figure 6). The data for the nine most cited journals show that there are some common characteristics in the journal specifics. In all cases, we observed clear spikes on day 0, as a lot of breaking medical research reflected on Wikipedia immediately, which confirms our previous findings. The next year shows a huge decline in the number of citations.

*Cochrane Database of Systematic Reviews* stands out as a very commonly cited source, with over 300 counts for day 0. Since this journal focuses on review articles and Wikipedia favors review articles over primary sources [19], this observation is not surprising. *PLOS One* stands out too, as it is very quickly reflected on Wikipedia. This multidisciplinary mega-journal was established in 2006; given that some articles require over 10 years to be reflected, the young age of *PLOS One* may have affected its peculiarity. Moreover, *PLOS One* is an immediate open-access journal, which is in line with many Wikipedia editors’ philosophy. Interestingly, although PNAS is also a multidisciplinary journal, albeit not with fully open access, it does not rank among the top journals in our study. PNAS seemed to lose some of its importance among the editors over time.

Interestingly, *Nature* and *Science* either reach Wikipedia relatively slower or are more systematically backlogged (editors add journal articles from the further past). These journals do not focus on medicine specifically, which may explain a flatter distribution: In other words, publishing in these journals sooner or later results in coverage by Wikipedia. This is not necessarily the case for journals dedicated solely to medicine. Articles in medicine-only journals tend to appear on Wikipedia nearly immediately or are quite unlikely to ever be reflected.

**Table 2:** Top 10 most cited journals in three time periods (cumulative). The total number of citations in an analyzed period is presented within parentheses.

Rank	2000 and later	2007 and later	2012 and later
1	Cochrane Database of Systematic Reviews (1388)	Cochrane Database of Systematic Reviews (1073)	Cochrane Database of Systematic Reviews (720)
2	The New England Journal of Medicine (1291)	The New England Journal of Medicine (731)	PLOS One (352)
3	The Lancet (1156)	PLOS One (616)	The New England Journal of Medicine (255)
4	The BMJ (921)	The BMJ (557)	Annals of Internal Medicine (200)
5	Proceedings of the National Academy of Sciences of the United States of America (888)	The Lancet (424)	The Lancet (196)
6	JAMA: The Journal of the American Medical Association (822)	JAMA: The Journal of the American Medical Association (422)	The BMJ (192)
7	Nature (802)	Nature (357)	JAMA: The Journal of the American Medical Association (153)
8	Science (739)	Proceedings of the National Academy of Sciences of the United States of America (335)	Nature (147)
9	PLOS One (620)	Annals of Internal Medicine (273)	Neurology (110)
10	Journal of Biological Chemistry (560)	Neurology (224)	Proceedings of the National Academy of Sciences of the United States of America (94)

Our final finding is based on a ranking of the top 60 journals most commonly cited in Wikipedia medical articles. The differences between our ranking and those of Journal Citation Reports (JCR) or Scientific Journal Ranking (SJR) would indicate the specifics of Wikipedia (what is likely to be cited by a medical journal can differ from what is typically cited on Wikipedia; for instance, reviews and meta-analyses are more likely to be reflected in Wikipedia). However, a direct comparison is difficult, as other rankings have different criteria for considering a journal as medical, and general science journals would have to be included in some way.

The top 30 most commonly cited journals are much more diverse in the number of citations, and thus, we decided to offer a ranking. However, for positions 30 to 60, we did not rank the positions and listed the journals alphabetically, as the differences among them were too small.

As noted above, changes in the number of Wikipedia citations are dynamic over time, including irregular spikes and visible periods of maturation after Wikipedia’s inception. Therefore, we propose a score for ranking, which accommodates the fast-changing environment of Wikipedia.

**Figure 7:**
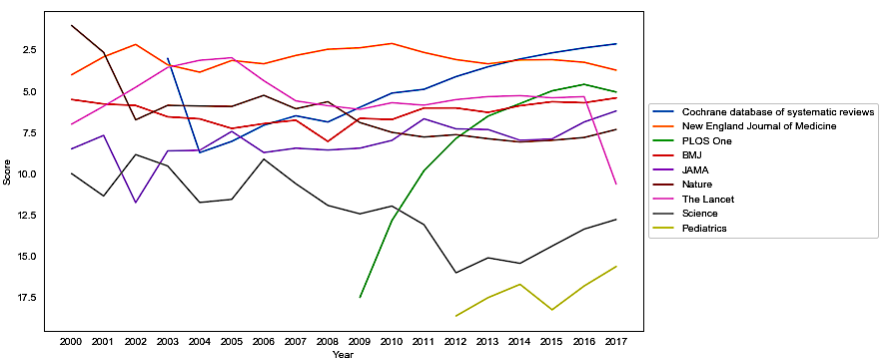
Journal citations over time.

We propose a score calculated as an exponential moving average of a year-to-year ranking based on the number of citations. Using ranking instead of the absolute number of citations smoothens out the spikes and influx of citations that followed the creation of Wikipedia (Figure 7). Next, a moving average smoothens out large yearly variability in the ranking position. We believe this construction of ranking fairly reflects the relative popularity of journals in Wikipedia.

Our ranking for the top 30 journals is proposed in Table 3. There is a lot of variability for journals in positions above 30. Interestingly, only a few journals were considered to be best as per Wikipedia. Our ranking has an advantage over JCR or SJR rankings: It addresses the problem of general journals receiving high values for articles that are not related to medicine, as only medicine-related citations matter in our ranking.

**Table 3:** Top 30 journals according to the study results.

Journal	Rank
The Cochrane Database of Systematic Reviews	1
The New England Journal of Medicine	2
PLOS One	3
The BMJ	4
JAMA: The Journal of the American Medical Association	5
Nature	6
The Lancet	7
Science	8
Pediatrics	9
Blood	10
Annals of Internal Medicine	11
Circulation	12
Neurology	13
BMJ Open	14
Journal of Clinical Oncology	15
Scientific Reports	16
Clinical Infectious Diseases	17
The Journal of Clinical Psychiatry	18
Nature Communications	19
Stroke	20
Emerging Infectious Diseases	21
Biochemical and Biophysical Research Communications	22
Neuropharmacology	23
Canadian Medical Association Journal	24
The Lancet Infectious Diseases	25
The American Journal of Medicine	26
Gastroenterology	27
International Journal of Cancer	28
Academic Emergency Medicine	29
Archives of Disease in Childhood	30

## Discussion

We found evidence of “recentism,” which refers to preferential citation of recently published journal articles in Wikipedia. Traditional high-impact medical and multidisciplinary journals were highly cited by Wikipedia, suggesting that Wikipedia medical articles have robust underpinnings. In keeping with the Wikipedia policy of citing reviews/secondary sources over primary sources, the *Cochrane Database of Systematic Reviews* was the most referenced journal, possibly due to an established systematic collaboration with Wikipedia [20].

Our study shows a ranking of journals according to their actual, practical usability in Wikipedia medical articles, which may be advantageous over journal self-descriptions or preconceptualized categorizations. It allows inclusion of general science and health journals and is an alternative, if not more reliable, measure of journal impact on popular knowledge based on decisions of the self-governed, peer-production community.

Our study has a few limitations. Our analysis was limited to the largest Wikipedia, the English-language one. It is possible that other language Wikipedias might have divergent patterns, as demonstrated by prior research [10]. Where possible, the most widely used unique identifier for scholarly journal articles—the digital object identifier (DOI)—was used [14]. However, some journal articles cited by Wikipedia did not have had an identifier. In such instances, where possible, journals were identified using the publication title, journal name, and Crossref application programming interface. Due to the presence of abbreviations, incomplete entries or identification of misspelling was not always possible, and therefore, some entries were missed. Books, regarded as secondary sources and thus likely to be preferentially cited on Wikipedia’s medical pages as per internal guidelines, were not included in this analysis. In addition, articles published before the year 2000 may have statistically worse coverage by Crossref and the DOI than contemporary articles. There was a bias in our results toward journals that cover clinical topics, because the *Journal of Medical Internet Research* and related journals were cited mainly by Wikipedia pages on eHealth/Health informatics. These pages were predominantly categorized under the fields of computing or technology instead of medicine. To minimize this limitation, future studies could begin with a list of biomedical journals and subsequently search where these studies are cited in Wikipedia, regardless of category. A notable strength of this study was the availability of robust date data for a large number of journal articles. Notably, it is estimated that 694,930 references supporting medical content were published on Wikipedia in 2017, including all kinds of sources [21].

Given the fact that Wikipedia editing increases information literacy [22-24] and that Wikipedia is increasingly adopted by academics [25], we believe that Wikipedia can be relied upon to supplement our knowledge about journal quality. Similar research for other disciplines is warranted.

## Appendix

Multimedia Appendix 1 Methods.

Multimedia Appendix 2 Top 200 journals.

## Abbreviations

COI: conflict of interest
DOI: digital object identifier
JCR: Journal Citation Reports
PNAS: Proceedings of the National Academy of Sciences
SJR: Scientific Journal Ranking
