# Supplementary material for: The Most Influential Medical Journals According to Wikipedia: Quantitative Analysis
Source: J Med Internet Res. 2019 Jan 18;21(1):e11429. doi: 10.2196/11429 (PMC6356187; doi:10.2196/11429)
Supplement: Multimedia Appendix 1 [file jmir_v21i1e11429_app1.pdf]

Methods appendix.

We evaluated the number of days between journal article publication and its citation in the English language Wikipedia, treating it as a proxy for information diffusion rate. For our analysis, we selected 39,561 medical articles on wiki, marked as “medical” by Wikipedia community [16] (as of October 10th, 2017).

In the following days, through wiki application programming interface (API) with help of pywikibot, we collected current version of the articles. Out of 39,561 Wikipedia articles 15,678 did not contain any journal references at the time of collection (October 2017).

For every article a list of journal citations was collected based on “cite journal” presence in “ref” tag. In the next step, we retrieved every revision of the articles in a monthly interval. The first revision in a every month was used.

Next every revision was scanned for the presence of every journal reference from the current version. The oldest revision with a given reference was treated as the time of Wikipedia citation.

In order to find publication date for every reference a query was made to Crossref API. The result was checked using Levenshtein distance between article titles and journal names. As many journal names are used in abbreviated form in Wikipedia references, a handmade dictionary of abbreviations was used (j = journal, eur = european, etc) along with text simplification (lower case, no punctuation) to increase the reliability of the method.

We found citations to 137,889 articles from over 15,000 journals, in 11,314 Wikipedia medical articles. We retrieved the dates of article inclusion on Wikipedia, as well as the date of publication from Crossref for 108,600 references. In 8,384 of these references the date of addition into Wikipedia preceded the official date of publication, which does not necessarily signify an error, but rather might refer to pre-prints.

To compute the ranking of the most cited journals a de-duplication procedure was conducted to make sure that different forms of the same journal name were properly attributed. This procedure was split into automated de-duplication and handmade dictionary. In the automated stage abbreviation dictionary was used. Next, for the 100 top cited journal we looked for the most similar journal names and created a list of synonyms for those journals by hand. Due to large similarities between some journal names full automation of this process was not possible.

The analysis was limited to the largest Wikipedia, the English language one. Where possible, the most widely used unique identifier for scholarly journal articles, the digital object identifier (doi), was used. However most journal articles cited by Wikipedia might not have had an identifier. In those instances, where possible, identification of journal was made using publication title, journal name and Crossref API. Due to abbreviations, incomplete entries or misspelling identification was not always possible and thus some entries were missed.
